# Risk factors for anastomotic leakage and its impact on long-term survival in left-sided colorectal cancer surgery

**DOI:** 10.1186/s12957-020-01968-8

**Published:** 2020-08-14

**Authors:** Marius Kryzauskas, Augustinas Bausys, Austeja Elzbieta Degutyte, Vilius Abeciunas, Eligijus Poskus, Rimantas Bausys, Audrius Dulskas, Kestutis Strupas, Tomas Poskus

**Affiliations:** 1 Clinic of Gastroenterology, Nephrourology and Surgery, Institute of Clinical Medicine, Faculty of Medicine, Vilnius University, Vilnius, Lithuania; 2 Faculty of Medicine, Vilnius University, Ciurlionio str. 21, 03101 Vilnius, Lithuania

**Keywords:** Colorectal cancer, Anastomotic leakage, Risk factors, Oncological outcomes, Overall survival, Disease-free survival

## Abstract

**Background:**

Anastomotic leakage (AL) significantly impairs short-term outcomes. The impact on the long-term outcomes remains unclear. This study aimed to identify the risk factors for AL and the impact on long-term survival in patients with left-sided colorectal cancer.

**Methods:**

Nine-hundred patients with left-sided colorectal carcinoma who underwent sigmoid or rectal resection were enrolled in the study. Risk factors for AL after sigmoid or rectal resection were identified, and long-term outcomes of patients with and without AL were compared.

**Results:**

AL rates following sigmoid and rectal resection were 5.1% and 10.7%, respectively. Higher ASA score (III–IV; OR = 10.54, *p* = 0.007) was associated with AL in patients undergoing sigmoid surgery on multivariable analysis. Male sex (OR = 2.40, *p* = 0.004), CCI score > 5 (OR = 1.72, *p* = 0.025), and T3/T4 stage tumors (OR = 2.25, *p* = 0.017) were risk factors for AL after rectal resection on multivariable analysis. AL impaired disease-free and overall survival in patients undergoing sigmoid (*p* = 0.009 and *p* = 0.001) and rectal (*p* = 0.003 and *p* = 0.014) surgery.

**Conclusion:**

ASA score of III–IV is an independent risk factor for AL after sigmoid surgery, and male sex, higher CCI score, and advanced T stage are risk factors for AL after rectal surgery. AL impairs the long-term survival in patients undergoing left-sided colorectal surgery.

## Introduction

Anastomotic leakage (AL) is one of the most devastating complications following colorectal resection for left-sided colorectal cancer (CRC) [1]. It leads to increased morbidity, mortality, treatment costs, and prolonged hospitalization. The AL rate varies between 6 and 12% after rectal resection and between 2 and 4% after sigmoid resection [2]. Male sex, elderly age, obesity, severe comorbidities (higher American Society of Anesthesiology (ASA) score), prolonged surgery time, perioperative blood transfusions, low anastomosis, and neoadjuvant chemoradiotherapy are proposed risk factors for AL [3, 4]. AL may occur in patients without any risk factors as well, and therefore, it remains a challenging issue in CRC surgery.

While AL has a negative impact on short-term surgical outcomes, the impact on long-term outcomes remains controversial. The study led by Karim et al. concluded that AL impairs overall survival (OS) and disease-free survival (DFS) [5]. In contrast, Crippa et al. reported similar outcomes in patients with or without AL in terms of OS, DFS, and local recurrence rates [6]. Therefore, the present study aimed to determine the impact of AL on the long-term outcomes in patients undergoing surgery for left-sided CRC and to identify the risk factors for AL after sigmoid and rectal resection.

## Materials and methods

### Ethics

Vilnius regional research ethics committee approval (no. 2019/3-116-608) was obtained before the study. The study was conducted according to the Declaration of Helsinki.

### Patients

**Fig. 1:**
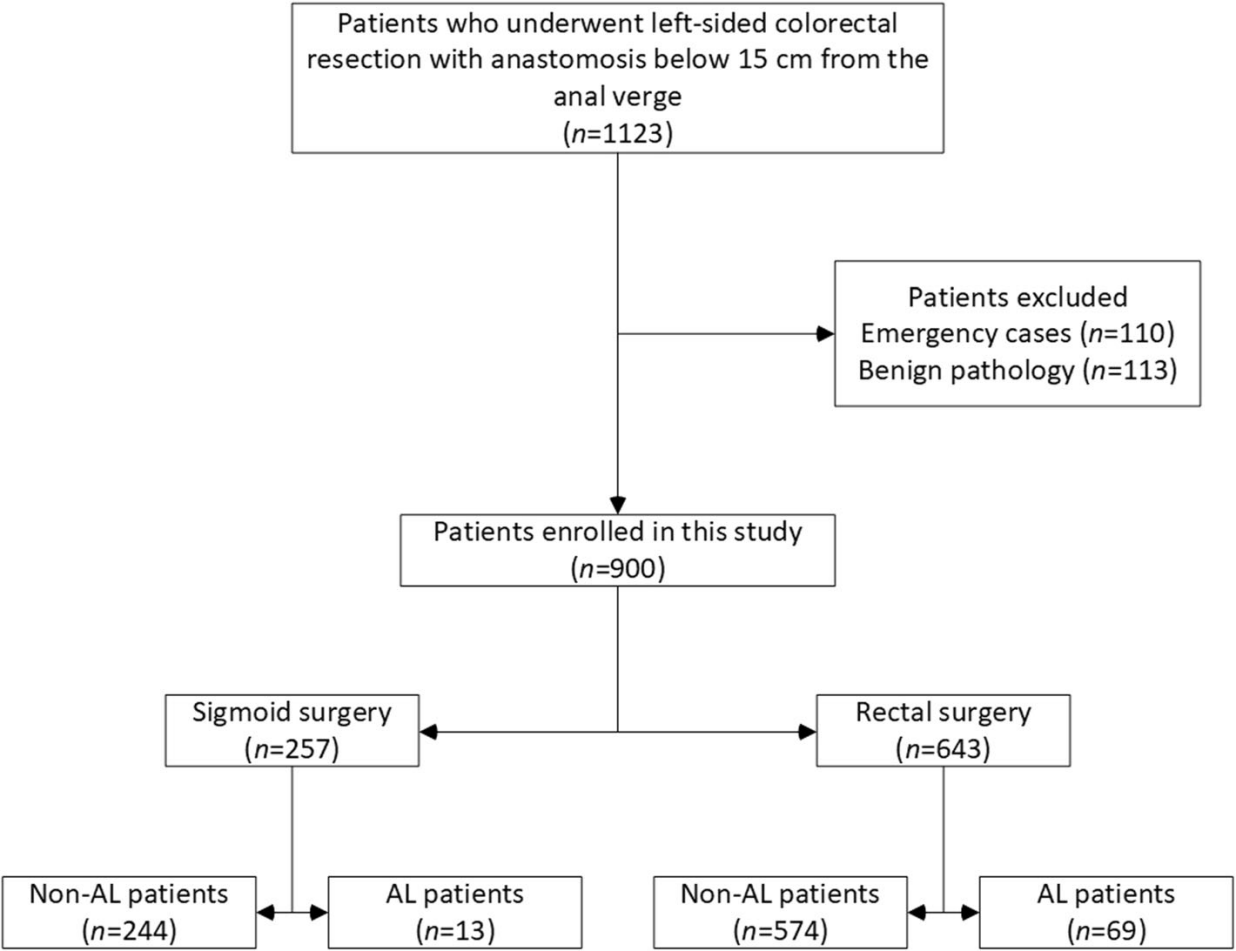
Flowchart of the patients selection process

All patients who underwent left-sided colorectal resection with a primary anastomosis below 15 cm from anal verge between January 2014 and December 2018 at two major gastrointestinal cancer treatment centers in Lithuania—Vilnius University Hospital Santaros Klinikos and National Cancer Institute—were screened for eligibility. Patients who underwent emergency surgery or those with a benign pathology were excluded. Finally, all patients who underwent elective colorectal resection with primary anastomosis for left-sided CRC were included in the study (Fig. 1).

### Data collection

All participants’ characteristics were obtained from the prospectively collected and maintained databases. They included age, gender, body mass index (BMI), American Society of Anesthesiologists (ASA) score, comorbidities, Charlson comorbidity index (CCI), history of neoadjuvant treatment, tumor localization, surgical approach (open surgery and minimally invasive surgery, laparoscopic surgery, hand-assisted laparoscopic surgery, natural orifice specimen extraction surgery, and transanal total mesorectal excision surgery (taTME)), the level of the anastomosis, diverting ileostomy, simultaneous operation, high or low ligation of the inferior mesenteric artery (IMA), results of the intraoperative air-leak test, postoperative complications including AL, and the data of follow-up including progression of the disease. Tumor stage was coded according to the TNM system as described in the Union Internationale Contre le Cancer/American Joint Committee on Cancer 8th edition.

### Study outcomes

The primary outcome of the study was overall survival (OS) in patients with or without AL. The secondary outcomes included disease-free survival (DFS), 30-day mortality, and the risk factors for AL.

OS was defined as the time from surgery to death. Data on survival and date of death were collected from the National Lithuanian Cancer registry and the National Lithuanian death registry. Mortality registration rates, from both resources, were over 98%. DFS was defined as the time from surgery to disease progression including local or distant recurrence.

### AL definition

AL was defined as a defect at the anastomotic area with a communication between the intra- and extra-luminal compartments. AL was confirmed by digital rectal examination, endoscopic evaluation, or radiologic tests with proven extravasation of rectal contrast or evidence of a peri-anastomotic fluid collection with pus or feculent aspirate [7].

### Statistical analysis

All statistical analysis was performed by statistical package SPSS 25.0 (SPSS, Chicago, IL, USA). Patients were grouped to those who developed AL (AL) and those who did not develop AL (non-AL). All data were checked for normality. Continuous variables were compared by a two-tailed *t* test, one-way ANOVA, or non-parametric tests where appropriate and expressed as means ± standard deviation or median with first (Q1) and third (Q3) quartiles. Categorical data were expressed as proportions with percentages and compared by the chi-square test and Fisher exact test. To identify independent variables associated with anastomotic leakage, all potential risk factors were included in subsequent multivariable logistic regression analyses. Kaplan-Meier method was used for OS analysis, and survival curves were compared by the log-rank test. Multivariable survival analysis was performed using the Cox proportional hazards model (hazard ratio and 95% confidence intervals). A *p* value of < 0.05 was considered to be significant in all statistical analyses.

## Results

### Study participants

A total of 900 patients with a mean age of 65 ± 10 years were included in the study. For further analysis, patients were divided into sigmoid and rectal surgery sub-groups based on a tumor location. The AL rate after sigmoid and rectal surgery was 5.1% (13 of 257) and 10.7% (69 of 643), respectively. Baseline data of the patients are included in Table 1.

**Table 1:** Basic characteristics of the sigmoid and rectal surgery groups

		Sigmoid surgery (*n* = 257)	Rectal surgery (*n* = 643)	*p* value
Age		65.4 ± 10.2	65.1 ± 10.9	0.172
BMI	< 30	152 (63.1%)	472 (77.6%)	0.001
	≥ 30	89 (36.9%)	136 (22.4%)	
Gender	Female	117 (45.5%)	300 (46.7%)	0.768
	Male	140 (54.5%)	343 (53.3%)	
ASA	I–II	160 (65.3%)	430 (70.1%)	0.167
	III–IV	85 (34.7%)	183 (29.9%)	
CCI	≤ 5	180 (70.0%)	470 (73.1%)	0.365
	> 5	77 (30.0%)	173 (26.9%)	
T stage	T0–T2	86 (33.5%)	215 (33.4%)	0.999
	T3–T4	171 (66.5%)	428 (66.6%)	
N stage	0	149 (59.4%)	392 (61.6%)	0.822
	1	72 (28.7%)	172 (27.0%)	
	2	30 (12.0%)	72 (11.3%)	
M stage	0	218 (84.8%)	592 (92.1%)	0.002
	1	39 (15.2%)	51 (7.9%)	
TNM stage	0	5 (1.9%)	7 (1.1%)	0.006
	1	66 (25.7%)	160 (24.9%)	
	2	71 (27.6%)	198 (30.8%)	
	3	75 (29.2%)	227 (35.3%)	
	4	40 (15.6%)	52 (7.9%)	
Approach of surgery	Open	78 (30.4%)	364 (56.6%)	0.001
	MI	179 (69.6%)	279 (43.4%)	
Postoperative complications	No	208 (80.9%)	413 (64.2%)	0.001
	Yes	49 (19.1%)	230 (35.8%)	
AL	No	244 (94.9%)	574 (89.3%)	0.007
	Yes	13 (5.1%)	69 (10.7%)	

*ASA* American Society of Anesthesiologists classification score, *CCI* Charlson comorbidity index score, *MI* minimally invasive, *AL* anastomotic leakage

### Risk factors for AL

Table 2 shows the univariate analysis of all potential risk factors for AL after sigmoid and rectal surgery. Higher ASA score (III–IV, *p* = 0.002) was associated with AL in patients undergoing sigmoid surgery, while male sex (*p* = 0.002), higher CCI score (> 5, *p* = 0.004), and advanced tumor stage (T3/T4, *p* = 0.031) was associated with AL in patients with rectal cancer.

**Table 2:** Univariate analysis of risk factors for postoperative AL in sigmoid and rectal surgery

		Sigmoid surgery			Rectal surgery		
		Non-AL	AL	*p* value	Non-AL	AL	*p* value
		(*n* = 244)	(*n* = 13)		(*n* = 574)	(*n* = 69)	
BMI	< 30	147 (96.7%)	5 (3.3%)	0.337	424 (89.8%)	48 (10.2%)	0.999
	≥ 30	83 (93.3%)	6 (6.7%)		122 (89.7%)	14 (10.3%)	
Gender	Female	111 (94.9%)	6 (5.1%)	0.999	280 (93.3%)	20 (6.7%)	0.002
	Male	133 (95.0%)	7 (5.0%)		294 (85.7%)	49 (14.3%)	
ASA	I–II	158 (98.8%)	2 (1.3%)	0.002	389 (90.5%)	41 (9.5%)	0.154
	III–IV	76 (89.4%)	9 (10.6%)		158 (86.3%)	25 (13.7%)	
CCI	≤ 5	172 (95.6%)	8 (4.4%)	0.538	430 (91.5%)	40 (8.5%)	0.004
	> 5	72 (93.5%)	5 (6.5%)		144 (83.2%)	29 (16.8%)	
Ischemic heart disease	Yes	13 (100%)	0 (0%)	0.999	26 (86.7%)	4 (13.3%)	0.551
	No	231 (94.7%)	13 (5.3%)		548 (89.4%)	65 (10.6%)	
Diabetes mellitus	Yes	28 (90.3%)	3 (9.7%)	0.197	54 (84.4%)	10 (15.6%)	0.200
	No	216 (95.6%)	10 (4.4%)		520 (89.8%)	59 (10.2%)	
History of CVD	Yes	8 (100%)	0 (0.0%)	0.999	17 (89.5%)	2 (10.5%)	0.999
	No	236 (94.8%)	13 (5.2%)		557 (89.3%)	67 (10.7%)	
Chronic renal failure	Yes	4 (100%)	0 (0.0%)	0.999	8 (88.9%)	1 (11.1%)	0.999
	No	240 (94.9%)	13 (5.1%)		566 (89.3%)	68 (10.7%)	
Neoadjuvant treatment	Yes	8 (88.9%)	1 (11.1%)	0.378	161 (88.0%)	22 (12.0%)	0.484
	No	236 (95.2%)	12 (4.8%)		413 (89.8%)	47 (10.2%)	
Approach of surgery	Open	71 (91.0%)	7 (9.0%)	0.069	320 (87.9%)	44 (12.1%)	0.247
	MI	173 (96.6%)	6 (3.4%)		254 (91.0%)	25 (9.0%)	
Anastomosis level from anal verge	≤ 5	20 (95.2%)	1 (4.8%)	0.999	155 (89.1%)	19 (10.9%)	0.137
	6–12				235 (86.4%)	37 (13.6%)	
	> 12	178 (93.7%)	12 (6.3%)		81 (94.2%)	5 (5.8%)	
Ileostomy	Yes	0 (0.0%)	1 (100%)	0.051	330 (88.5%)	43 (11.5%)	0.519
	No	244 (95.3%)	12 (4.7%)		244 (90.4%)	26 (9.6%)	
T stage	T0–T2	84 (97.7%)	2 (2.3%)	0.230	200 (93.0%)	15 (7%)	0.031
	T3–T4	160 (93.6%)	11 (6.4%)		374 (87.4%)	54 (12.6%)	
N stage	0	143 (96%)	6 (4.0%)	0.253	357 (91.1%)	35 (8.9%)	0.130
	1	70 (97.2%)	2 (2.8%)		149 (86.6%)	23 (13.4%)	
	2	27 (90.0%)	3 (10%)		61 (84.7%)	11 (15.3%)	
M stage	0	209 (95.9%)	9 (4.1%)	0.117	531 (89.7 %)	61 (10.3 %)	0.238
	1	35 (89.7%)	4 (10.3%)		43 (84.3 %)	8 (15.7 %)	
TNM stage	0	5 (100%)	0 (0.0%)	0.221	6 (85.7%)	1 (14.3%)	0.568
	1	64 (97.0%)	2 (3.0%)		147 (91.9%)	13 (8.1%)	
	2	68 (95.8%)	3 (4.2%)		178 (89.9%)	20 (10.1%)	
	3	72 (96.0%)	3 (4%)		200 (88.1%)	27 (11.9%)	
	4	35 (87.5%)	5 (12.5%)		43 (84.3%)	8 (15.7%)	
Ligation of IMA	High	189 (95.9%)	8 (4.1%)	0.165	442 (88.4%)	58 (11.6%)	0.265
	Low	50 (90.9%)	5 (9.1%)		117 (92.1%)	10 (7.9%)	
Simultaneous operation	Yes	20 (90.9%)	2 (9.1%)	0.308	53 (85.5%)	9 (14.5%)	0.286
	No	224 (95.3%)	11 (4.7%)		521 (89.7%)	60 (10.3%)	
Air-water test	Yes	121 (96.8%)	4 (3.2%)	0.255	454 (88.7%)	58 (11.3%)	0.429
	No	119 (93.0%)	9 (7.0%)		116 (91.3%)	11 (8.7%)	

*BMI* body mass index, *ASA* American Society of Anesthesiologists classification score, *CCI* Charlson comorbidity index score, *CVD* cardiovascular disease, *MI* minimally invasive, *IMA* inferior mesenteric artery, *AL* anastomotic leakage

Further, the multivariable analysis confirmed a higher ASA score (III–IV; OR = 10.539; *p* = 0.007) as an independent risk factor for AL after sigmoid surgery (Table 3). The same analysis confirmed male sex (OR = 2.403, *p* = 0.004), higher CCI score (> 5, OR = 1.720, *p* = 0.025), and advanced tumor stage (T3/4, OR = 2.250; *p* = 0.017) were among risk factors for AL after rectal surgery (Table 4).

**Table 3:** Multivariable analysis of risk factors for postoperative AL in sigmoid surgery

	Risk factor	Odds ratio	95% CI	*p* value
Age		0.962	0.878–1.054	0.632
Gender	Male	0.834	0.179–3.882	0.784
BMI	> 30	1.519	0.283–8.153	0.119
ASA	III–IV	10.539	1.292–85.976	0.007
CCI	> 5	0.348	0.029–4.199	0.928
Diabetes mellitus	Yes	2.150	0.285–16.233	0.095
Surgery type	Palliative	1.726	0.052–57.273	0.601
Neoadjuvant treatment	Yes	9.657	0.269–346.401	0.307
Anastomosis type	Stapled	0.901	0.092–8.821	0.316
Ligation of IMA	High	0.670	0.093–4.848	0.081
Air-water test	No	1.084	0.060–19.593	0.187
Simultaneous operation	Yes	1.318	0.088–19.748	0.904
T stage	T3–T4	0.887	0.122–6.470	0.408
Approach of surgery	Open	0.438	0.070–2.731	0.079

*BMI* body mass index, *ASA* American Society of Anesthesiologists classification score, *CCI* Charlson comorbidity index score, *IMA* inferior mesenteric artery, *AL* anastomotic leakage

**Table 4:** Multivariable analysis of risk factors for postoperative AL in rectal surgery

	Risk factor	Odds ratio	95% CI	*p* value
Gender	Male	2.403	1.204–4.797	0.004
Age		0.994	0.962–1.026	0.307
BMI	> 30	0.858	0.389–1.894	0.495
ASA	III–IV	1.346	0.635–2.854	0.156
CCI	> 5	1.720	0.759–3.898	0.025
Diabetes mellitus	Yes	1.297	0.478–3.522	0.155
Ischemic heart disease	Yes	0.933	0.250–3.487	0.303
Cerebrovascular disease	Yes	1.090	0.185–6.432	0.644
Surgery type	Palliative	0.606	0.059–6.273	0.980
Neoadjuvant treatment	Yes	1.430	0.645–3.170	0.260
Anastomosis type	Stapled	1.310	0.125–13.727	0.809
Ligation of IMA	High	2.345	0.939–5.856	0.167
Air-water test	No	1.339	0.529–3.392	0.350
Ileostomy	No	0.884	0.405–1.930	0.749
Simultaneous operation	Yes	1.188	0.436–3.237	0.450
T stage	T3–4	2.250	1.052–4.815	0.017
Approach of surgery	Open	0.633	0.316–1.269	0.186
Anastomosis level from anal verge	< 5	3.286	0.933–11.569	0.064
Anastomosis level from anal verge	5–12	2.629	0.636–10.868	0.182

*BMI* body mass index, *ASA* American Society of Anesthesiologists classification score, *CCI* Charlson comorbidity index score, *IMA* inferior mesenteric artery, *AL* anastomotic leakage

### AL and 30- and 90-day mortality

The 30-day mortality rate was higher in patients with AL in the sigmoid (15.4% vs 0%, *p* = 0.002) and rectal (5.8% vs 1%, *p* = 0.016) surgery sub-groups. Similarly, 90-day mortality rate remained higher in leaking patients (sigmoid 15.4% vs 1.6%, *p* = 0.032; rectal 8.7% vs 2.1%, *p* = 0.008).

### AL and long-term outcomes

**Fig. 2:**
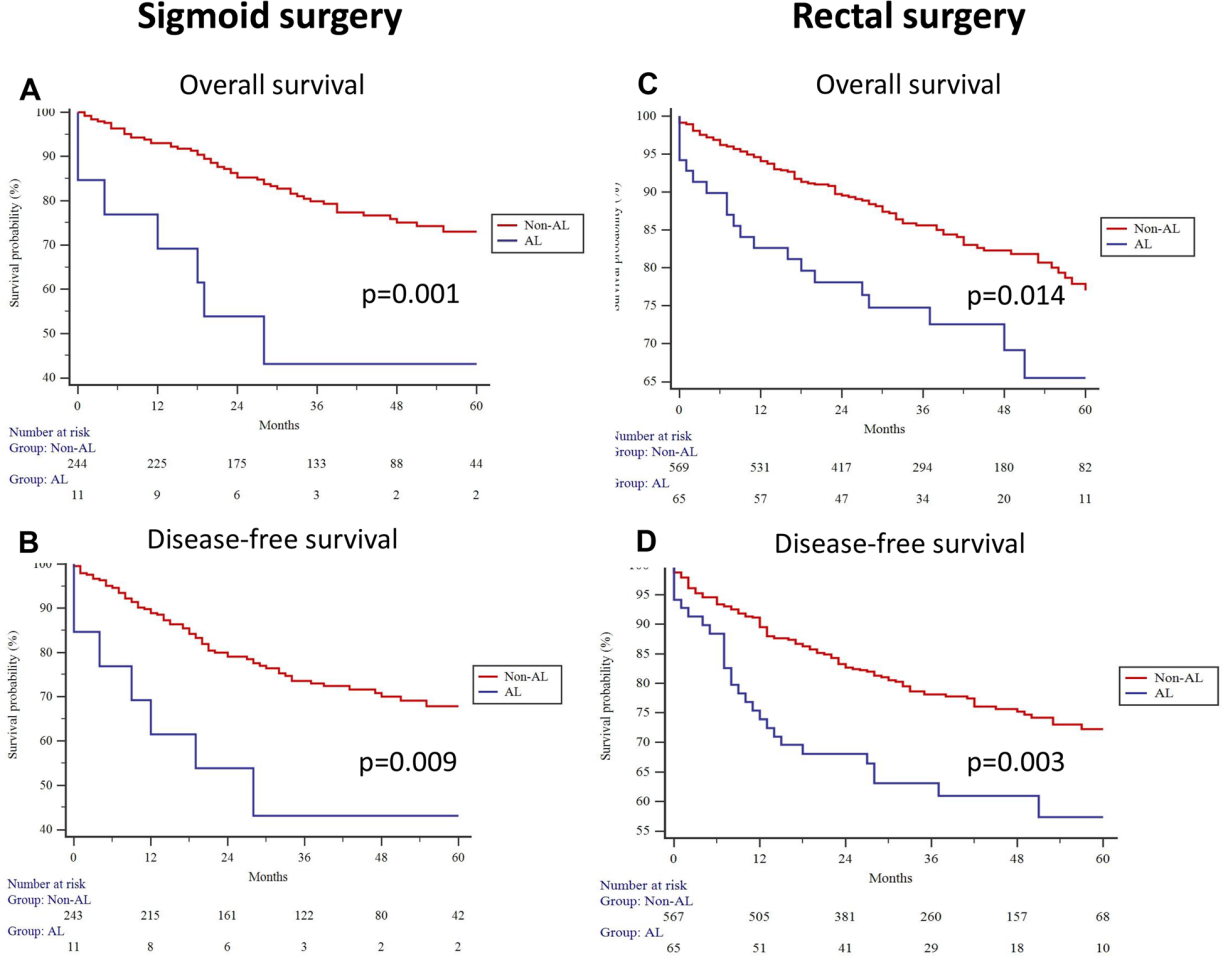
Overall and disease-free survival in sigmoid and rectal surgery

The median time of follow-up was 38 (Q1 22; Q3 53) months. The AL after sigmoid surgery impaired OS and DFS (Fig. 2a, b). Similarly, the AL impaired OS and DFS (Fig. 2c, d) after rectal surgery.

Further, AL was adjusted for the stage of the disease, gender, age, and comorbidities (CCI score) by COX regression analysis in the study cohort. After, the AL remained a significant factor for impaired OS (HR (95% CI) 1.53 (1.01–2.32), *p* = 0.041) and DFS (HR (95% CI) 1.51 (1.05–2.19), *p* = 0.026) (Table 5).

**Table 5:** Cox regression (multivariable) analysis for overall and disease-free survival in the study cohort

		Overall survival		Disease-free survival	
		HR (95% CI)	*p*	HR (95% CI)	*p*
Anastomotic leakage	No	1 (reference)		1 (reference)	
	Yes	1.53 (1.01–2.32)	0.041	1.51 (1.05–2.19)	0.026
Stage of disease	I	1 (reference)		1 (reference)	
	II	1.26 (0.72–2.20)	0.403	1.52 (0.93–2.48)	0.090
	III	2.28 (1.38–3.78)	0.001	2.94 (1.88–4.59)	0.001
	IV	5.87 (3.26–10.56)	0.001	6.04 (3.51–10.38)	0.001
Gender	Male	1 (reference)		1 (reference)	
	Female	1.03 (0.76–1.39)	0.832	0.95 (0.73–1.23)	0.714
Age (years)	≤ 55	1 (reference)		1 (reference)	
	56–70	1.15 (0.71–1.85)	0.566	1.02 (0.70–1.49)	0.889
	≥ 71	1.90 (1.16–3.11)	0.010	1.15 (0.76-1.73)	0.498
Comorbidities by CCI	0–5	1 (reference)		1 (reference)	
	≥ 6	2.48 (1.64–3.74)	0.001	2.14 (1.48–3.10)	0.001

*CCI* Charlson comorbidity index score

## Discussion

Our study demonstrated that AL impairs long-term outcomes of the patients undergoing surgery for sigmoid and rectal cancer. Severe comorbidities, male sex, and advanced tumor stage are the risk factors for AL.

Several recent studies investigated the risk factors for AL because the identification of high-risk patients and avoidance of anastomosis in these patients could improve treatment outcomes [8–12]. Previously, studies demonstrated male gender as a risk factor for AL after rectal surgery, and our results were consistent with these findings [3, 8, 9, 13, 14]. Male gender is thought to increase the AL rate because of more technically demanding surgery in the narrow and deeper pelvis of men [13]. There is a possibility that hormonal functions may impact anastomotic healing as well [15, 16]. The advanced stage of tumor also makes surgery more technically challenging, and it was confirmed as another risk factor for AL by our study. Interestingly, we did not find a higher AL rate in patients receiving low anastomosis. These findings are conflicting with some previous reports indicating a higher risk for low anastomoses [3, 17]. Although, in our results, there was a strong tendency for higher AL rate in low anastomoses (≤ 5 cm (10.9%) vs 6–12 cm (13.6%) vs > 12 cm (5.8 %), *p* = 0.137), and it might be that our study was underpowered to detect significant differences because of the relatively small sample size.

Lower anastomoses may be secured by diverting ileostomy. However, the evidence on the impact of ileostomy on preventing the leak or reducing the symptoms is conflicting. Two meta-analyses concluded that stoma reduces the rate of AL following low anterior resection [12, 18]. In contrast, our study did not confirm that ileostomy prevents AL. This finding is consistent with some previous studies [19, 20]. A temporary ileostomy may not prevent the AL but rather diminish its symptoms and consequences. Further, the true rate of AL in patients receiving ileostomy may be underestimated because usually asymptomatic patients do not undergo testing for anastomosis integrity at the early postoperative period [21, 22]. Similarly, in our study, asymptomatic patients underwent anastomosis integrity testing just before the ileostomy closure; thus, some cases of AL in patients who receive ileostomy might have been underestimated as well. Therefore, further studies are required to clarify the role of ileostomy in the prevention of the AL.

The existing data on AL impact on the long-term outcomes are conflicting as well. A recent study from the Mayo Clinic revealed similar OS, DFS, and local recurrence rates between patients with or without AL [6]. Propensity score-matched analysis by Sueda et al. also demonstrated a similar OS rate in AL and non-AL patients, except the higher rate of local recurrence in case of leakage [23]. In contrast, the previous meta-analysis by Bashir et al. concluded that patients with AL have a lower 5-year OS of 58% compared with 73% in non-leaking patients [24]. Moreover, the negative impact of AL on OS was indicated by Yang et al. and a large Scandinavian cohort study by Stormark et al. [25, 26]. Our study confirmed the impaired OS and DFS in patients suffering from AL, and there is a rationale for such findings. First, AL may lead to an increased rate of local recurrence because of cancer cell implantation and progression at the inflamed leaking anastomotic site [27, 28]. Despite AL occurs after surgical tumor removal, several viable tumor cells remain intraluminally, proximally, and distally to cancer sites [29]. These cells were identified after the rectal wash-out or were washed-out from histologically tumor-free stapled doughnuts [30, 31]. The pre-clinical model confirms these intraluminal cancer cells can implant at the anastomotic site and initiate tumor growth in experimental animals [32]. Additionally, the leakage results in a local inflammation, which may further contribute to the increased risk of tumor cell implantation and proliferation at the anastomotic site [33]. Moreover, the AL is associated with an increased systemic inflammatory response as shown by increased levels of CRP, and such condition may be related to the development and progression of the malignancy [34, 35]. AL is also associated with the delay or omission of the adjuvant chemotherapy. Therefore, AL may have a negative impact on long-term outcomes, especially in patients with the advanced stage of the disease, where adjuvant chemotherapy is necessary [36–39].

The present study has some limitations, including the retrospective design of the study. However, a considerable sample size, multicenter approach, and significant national registry-based long-term follow-ups increase the power of the study to demonstrate that AL is associated with impaired long-term outcomes in patients undergoing surgery for left-sided CRC. Future research is needed to find strategies to reduce or prevent the rate of AL in such patients [40].

## Conclusion

ASA score of III–IV is an independent risk factor for AL after sigmoid surgery, and male sex, higher CCI score, and advanced tumor stage are among risk factors for AL after rectal surgery. AL impairs the long-term survival in patients undergoing left-sided colorectal surgery.

## Supplementary information

**Additional file 1.** STROBE Statement.

## Data Availability

The datasets used and/or analyzed during the current study are available from the corresponding author on reasonable request.

## References

[1] Park JS, Huh JW, Park YA, et al. Risk factors of anastomotic leakage and long-term survival after colorectal surgery. Medicine. 2016;95(8). 10.1097/MD.0000000000002890 PMC4779025 26937928

[2] Leichtle SW, Mouawad NJ, Welch KB, Lampman RM, Cleary RK (2012). Risk factors for anastomotic leakage after colectomy. Dis Colon Rectum.

[3] Qu H, Liu Y (2015). Bi D-s. Clinical risk factors for anastomotic leakage after laparoscopic anterior resection for rectal cancer: a systematic review and meta-analysis. Surg Endosc.

[4] Hu M-H, Huang R-K, Zhao R-S, Yang K-L, Wang H (2017). Does neoadjuvant therapy increase the incidence of anastomotic leakage after anterior resection for mid and low rectal cancer? A systematic review and meta-analysis. Colorectal Disease: The Official Journal of the Association of Coloproctology of Great Britain and Ireland.

[5] Karim A, Cubas V, Zaman S, Khan S, Patel H, Waterland P. Anastomotic leak and cancer-specific outcomes after curative rectal cancer surgery: a systematic review and meta-analysis. Techniques in Coloproctology. 2020. 10.1007/s10151-020-02153-5 32206962

[6] Crippa J, Duchalais E, Machairas N, Merchea A, Kelley SR, Larson DW. Long-term oncological outcomes following anastomotic leak in rectal cancer surgery. Diseases of the Colon and Rectum. 2020. 10.1097/DCR.0000000000001634 32109914

[7] Rahbari NN, Weitz J, Hohenberger W (2010). Definition and grading of anastomotic leakage following anterior resection of the rectum: a proposal by the International Study Group of Rectal Cancer. Surgery..

[8] Rullier E, Laurent C, Garrelon JL, Michel P, Saric J, Parneix M (1998). Risk factors for anastomotic leakage after resection of rectal cancer. Br J Surg.

[9] Poon RT, Chu KW, Ho JW, Chan CW, Law WL, Wong J (1999). Prospective evaluation of selective defunctioning stoma for low anterior resection with total mesorectal excision. World J Surg.

[10] Law WI, Chu KW, Ho JW, Chan CW (2000). Risk factors for anastomotic leakage after low anterior resection with total mesorectal excision. Am J Surg.

[11] Mäkelä JT, Kiviniemi H, Laitinen S (2003). Risk factors for anastomotic leakage after left-sided colorectal resection with rectal anastomosis. Dis Colon Rectum.

[12] Wu S-W, Ma C-C, Yang Y (2014). Role of protective stoma in low anterior resection for rectal cancer: a meta-analysis. World J Gastroenterol: WJG.

[13] Matthiessen P, Hallböök O, Andersson M, Rutegård J, Sjödahl R (2004). Risk factors for anastomotic leakage after anterior resection of the rectum. Colorectal Disease: The Official Journal of the Association of Coloproctology of Great Britain and Ireland.

[14] Buchs NC, Gervaz P, Secic M, Bucher P, Mugnier-Konrad B, Morel P (2008). Incidence, consequences, and risk factors for anastomotic dehiscence after colorectal surgery: a prospective monocentric study. Int J Color Dis.

[15] Ba ZF, Yokoyama Y, Toth B, Rue LW, Bland KI, Chaudry IH (2004). Gender differences in small intestinal endothelial function: inhibitory role of androgens. Am J Physiol Gastrointest Liver Physiol.

[16] Sah BK, Chen M-M, Peng Y-B (2009). Does testosterone prevent early postoperative complications after gastrointestinal surgery?. World J Gastroenterol: WJG.

[17] Choi DH, Hwang JK, Ko YT (2010). Risk factors for anastomotic leakage after laparoscopic rectal resection. Journal of the Korean Society of Coloproctology.

[18] Montedori A, Cirocchi R, Farinella E, Sciannameo F, Abraha I (2010). Covering ileo- or colostomy in anterior resection for rectal carcinoma. The Cochrane Database of Systematic Reviews.

[19] Wong NY, Eu KW (2005). A defunctioning ileostomy does not prevent clinical anastomotic leak after a low anterior resection: a prospective, comparative study. Dis Colon Rectum.

[20] Salamone G, Licari L, Agrusa A (2016). Usefulness of ileostomy defunctioning stoma after anterior resection of rectum on prevention of anastomotic leakage A retrospective analysis. Ann Ital Chir.

[21] Bausys A, Kuliavas J, Dulskas A (2019). Early versus standard closure of temporary ileostomy in patients with rectal cancer: a randomized controlled trial. J Surg Oncol.

[22] Lee KH, Kim HO, Kim JS, Kim JY (2019). Prospective study on the safety and feasibility of early ileostomy closure 2 weeks after lower anterior resection for rectal cancer. Annals of Surgical Treatment and Research.

[23] Sueda T, Tei M, Yoshikawa Y (2020). Prognostic impact of postoperative intra-abdominal infections after elective colorectal cancer resection on survival and local recurrence: a propensity score-matched analysis. Int J Color Dis.

[24] Bashir Mohamed K, Hansen CH, Krarup P-M, Fransgård T, Madsen MT, Gögenur I (2020). The impact of anastomotic leakage on recurrence and long-term survival in patients with colonic cancer: a systematic review and meta-analysis. European Journal of Surgical Oncology: The Journal of the European Society of Surgical Oncology and the British Association of Surgical Oncology.

[25] Yang J, Chen Q, Jindou L, Cheng Y. The influence of anastomotic leakage for rectal cancer oncologic outcome: a systematic review and meta-analysis. J Surg Oncol. 10.1002/jso.25921 32243581

[26] Stormark K, Krarup P-M, Sjövall A, et al. Anastomotic leak after surgery for colon cancer and effect on long-term survival. Colorectal Disease. 2020;. 10.1111/codi.14999 32012414

[27] Skipper D, Jeffrey MJ, Cooper AJ, Alexander P, Taylor I (1989). Enhanced growth of tumour cells in healing colonic anastomoses and laparotomy wounds. Int J Color Dis.

[28] McGregor JR, Galloway DJ, George WD (1992). Intra-luminal tumour cells and peri-anastomotic tumour growth in experimental colonic surgery. European Journal of Surgical Oncology: The Journal of the European Society of Surgical Oncology and the British Association of Surgical Oncology.

[29] Umpleby HC, Fermor B, Symes MO, Williamson RC (1984). Viability of exfoliated colorectal carcinoma cells. Br J Surg.

[30] Jenner DC, de Boer WB, Clarke G, Levitt MD (1998). Rectal washout eliminates exfoliated malignant cells. Dis Colon Rectum.

[31] Gertsch P, Baer HU, Kraft R, Maddern GJ, Altermatt HJ (1992). Malignant cells are collected on circular staplers. Dis Colon Rectum.

[32] Kluger Y, Galili Y, Yossiphov J, Shnaper A, Goldman G, Rabau M (1998). Model of implantation of tumor cells simulating recurrence in colonic anastomosis in mice. Dis Colon Rectum.

[33] Terzić J, Grivennikov S, Karin E, Karin M (2010). Inflammation and colon cancer. Gastroenterology.

[34] Poskus E, Karnusevicius I, Andreikaite G, Mikalauskas S, Poskus T, Strupas K (2015). C-reactive protein is a predictor of complications after elective laparoscopic colorectal surgery: five-year experience. Wideochirurgia I Inne Techniki Maloinwazyjne = Videosurgery and Other Miniinvasive Techniques.

[35] Lu H, Ouyang W, Huang C (2006). Inflammation, a key event in cancer development. Molecular cancer research: MCR.

[36] Kim IY, Kim BR, Kim YW (2015). The impact of anastomotic leakage on oncologic outcomes and the receipt and timing of adjuvant chemotherapy after colorectal cancer surgery. International Journal of Surgery (London, England).

[37] Jung SH, Yu CS, Choi PW (2008). Risk factors and oncologic impact of anastomotic leakage after rectal cancer surgery. Dis Colon Rectum.

[38] Kube R, Mroczkowski P, Granowski D (2010). Anastomotic leakage after colon cancer surgery: a predictor of significant morbidity and hospital mortality, and diminished tumour-free survival. European Journal of Surgical Oncology: The Journal of the European Society of Surgical Oncology and the British Association of Surgical Oncology.

[39] Eberhardt JM, Kiran RP, Lavery IC (2009). The impact of anastomotic leak and intra-abdominal abscess on cancer-related outcomes after resection for colorectal cancer: a case control study. Dis Colon Rectum.

[40] Kryzauskas M, Poskus E, Dulskas A (2020). The problem of colorectal anastomosis safety. Medicine..

